# Impact of the Infection Period Distribution on the Epidemic Spread in a Metapopulation Model

**DOI:** 10.1371/journal.pone.0009371

**Published:** 2010-02-26

**Authors:** Elisabeta Vergu, Henri Busson, Pauline Ezanno

**Affiliations:** 1 UR341 Mathématiques et Informatique Appliquées, INRA, Jouy-en-Josas, France; 2 UMR1300 Bio-agression, Epidémiologie et Analyse de Risques, INRA, Nantes, France; 3 UMR1300 Bio-agression, Epidémiologie et Analyse de Risques, ENVN, Nantes, France; University of Nottingham, United Kingdom

## Abstract

Epidemic models usually rely on the assumption of exponentially distributed sojourn times in infectious states. This is sometimes an acceptable approximation, but it is generally not realistic and it may influence the epidemic dynamics as it has already been shown in one population. Here, we explore the consequences of choosing constant or gamma-distributed infectious periods in a metapopulation context. For two coupled populations, we show that the probability of generating no secondary infections is the largest for most parameter values if the infectious period follows an exponential distribution, and we identify special cases where, inversely, the infection is more prone to extinction in early phases for constant infection durations. The impact of the infection duration distribution on the epidemic dynamics of many connected populations is studied by simulation and sensitivity analysis, taking into account the potential interactions with other factors. The analysis based on the average nonextinct epidemic trajectories shows that their sensitivity to the assumption on the infectious period distribution mostly depends on 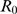, the mean infection duration and the network structure. This study shows that the effect of assuming exponential distribution for infection periods instead of more realistic distributions varies with respect to the output of interest and to other factors. Ultimately it highlights the risk of misleading recommendations based on modelling results when models including exponential infection durations are used for practical purposes.

## Introduction

The use of mathematical models in the study of epidemic dynamics, their mechanisms and their prevention and control provided valuable insights and contributed to draw up the global picture of epidemics occurrence, patterns and management [1]. Therefore, considering the impact of mathematical models in epidemiology, it is important to build them on realistic assumptions.

Most often, in mathematical models for epidemic spread, sojourn times in infectious states are exponentially distributed, which means that the probability of recovery per unit of time is constant, regardless of the time elapsed since infection. This classical assumption is routinely used for mathematical tractability-based reasons. Although this is sometimes an acceptable approximation, it is not realistic in general.

Several papers have explored the impact of more realistic non-exponentially distributed disease stages on the epidemic dynamics (summarized by various criteria, such as basic reproductive number (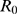), extinction probability, speed of propagation, epidemic burden, intensity of epidemic peak, etc) in single populations [2]–[5].

Most of these studies were motivated by the epidemiology of childhood viral diseases, such as measles, in order to reproduce their observed persistence patterns exhibiting localized extinctions. Since models making the assumption of exponential distributed infectious times were not able to mimic these observed patterns, Keeling and Grenfell [3], [6] tested constant sojourn times and provided simulations characterized by increasing persistence and therefore closer to observations. Independently, theoretical arguments were provided by Lloyd [7], [8] supporting the idea that constant infectious periods had a destabilizing effect on the global dynamics leading more often to extinction. This noticeable contradiction was discussed by Keeling and Grenfell [9] who proposed as a possible explanation the fact that parameters have to be updated with respect to the model, when the same observed phenomenon was described by different models. These authors argued that the results provided by theoretical studies were not realistic since in these works parameters were not updated when changing the model.

Recent papers in theoretical epidemiology have explored the question of 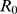 estimation from data with respect to other modelling assumptions, especially in the early phase of an epidemic [4], [10]–[13]. Their authors emphasized that caution has to be paid when calculating 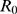 from estimations of the initial growth rate, since the equation relating these two parameters varies with respect to the distribution of the infection period.

Another category of modelling studies [14]–[19] have focused on epidemic dynamics in a metapopulation context, an important framework to explore when attempting to understand epidemic dynamics at a large scale [20]–[24]. Indeed, human or animal populations are not isolated; they influence each other (by exchanging individuals, for instance). Therefore, pathogens spread is the result of the complex interplay between intra-population events and inter-population interactions.

Despite the existence of numerous studies on these topics, less attention has been paid to connect both aspects, non-exponentially distributions for infectious periods and metapopulation global dynamics. The metapopulation context can be illustrated, for instance, by the transmission of an animal infection disease in a group of connected farms. Since models are often used to test the effectiveness of various interventions implemented at the scale of a region including many herds and since the assumption on the infectious stage distribution could affect model outcomes, it is important to correctly assess its effect.

Here, we focus on the analytical and computer-based exploration of different consequences of the introduction of non exponentially distributed sojourn times in epidemic models developed for metapopulation contexts. As already noticed, this is a question of non negligible importance, especially when models are built to serve as predictive tools. Indeed, the consequences of non realistic or inadequate modelling choices could provide biased results and hence orient towards inappropriate recommendations. Besides metapopulation aspects, our study differs from recent work focused on 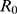 estimation as far as we adopt a different point of view on data. Whereas these papers [10], [12], [13] are interested in the estimation of 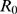 from data in the early stage of an outbreak, when little information about a disease is available, and focus on the relationship between 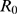 and the observed growth rate, we address situations where some knowledge about average epidemiological parameters of a disease is available and when the modelling is used to predict propagation at different time horizons. Indeed, such a situation can occur when information such as mean and range of infection duration are available based on expert opinion, but few data was collected. As an example, it was shown that for the contagious bovine pleuropneumonia, a respiratory disease of cattle exhibiting very diverse clinical patterns, constant infection durations were more appropriate than exponentially distributed ones for describing experimental data [25].

In this study, we explore possible discrepancies in forecasted dynamics in relation to model assumptions. More precisely, we are interested in: (i) what would happen (in terms of global criteria such as global epidemic burden, epidemic duration, extinction) if the infectious period was constant or gamma instead of exponentially distributed in a metapopulation context, when the epidemic is described by a 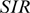 (Susceptible-Infectious-Removed) model in each patch and infection spreads between patches as a consequence of individuals movements; (ii) how the differences between these global criteria calculated for couples of distributions of the infectious period would vary with respect to 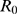, the transmission rate, the mean infection duration, the intensity of flows between patches and the network structure in a stochastic metapopulation model (consisting of 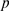 × 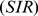 local models). Our approach is decomposed in two steps. First we focus on analytical developments of a specific criterion, the probability of early extinction, in single and two coupled populations. Analytical calculation is dedicated to the impact of the distribution of infectious period on the probability of no secondary infections and of extinction after 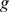 generations by extending the work of Keeling and Grenfell [3] to the case of gamma distribution in single populations. The two-population case is also explored concerning the probability that an infectious individual will cause no secondary cases. Second, since analytical explorations are intractable for more than two populations, we tackle the 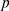-population case, where populations are connected through various networks and with different coupling intensities, by simulations using event-driven and individual-based approaches and statistical analysis of simulated data. We enlarge the framework by focusing on other aspects of an epidemic (such as epidemic size and duration, etc) in addition to the probability of early extinction. A sensitivity analysis (conducted through an analysis of variance) of various epidemic outputs (expressed as differences between outputs under different assumptions on the infectious period distribution) with respect to input factors such as 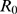 and network topology is performed.

Our paper is structured in several parts as follows: we first present the basic mathematical formulation of epidemic models we use, the main concepts necessary to the analytical explorations and the main lines of the sensitivity analysis. In the theoretical part of the results, analytical expressions of probabilities of early extinction and of extinction after 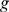 generations for single populations and two coupled populations are derived. In the applied subsection of results, computer-based explorations are performed for studying the sensitivity of the effects of infection period distribution to the input factors in a metapopulation framework. A general discussion is provided in the last section.

Throughout the article terms *patch* and *population* are used as synonyms to designate a local community with homogeneous contacts.

## Methods

State variables and transition probabilities of stochastic models are first described. Then, we define probabilities of interest in single and two-population models. Finally, we provide details on simulations performed for models including more than two populations and on statistical analysis of simulated data. Important parameters, variables and functions used throughout the paper are defined in Table 1.

**Table 1:** Summary of important parameters, variables and functions.

*Name*	*Expression*	*Definition*
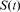		Number of susceptibles at 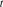
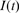		Number of infecteds at 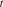
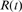		Number of recovereds at 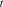
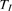 (day)		R.v.* infectious period
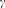 (day 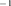 )		Recovery rate
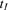 (day)	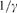	Mean of 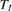
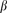 (day 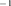 )		Transmission rate
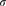 (day 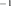 )		Intensity of migration
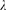	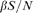	Rate of generation of new cases
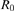	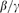	Basic reproductive number
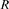	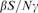	Effective reproductive number
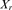		R.v.* number of secondary cases generated by an infectious individual
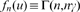	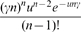	Probability density function of 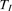
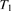 (day)		Mixed r.v.* time spent in the first population by an individual during his infectious period in a 2-population model
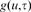	cf. eqs. (4) and (14)	Continuous component of 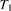
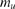	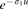	Mass component of 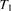
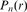	cf. eqs. (3) and (9)	Probability of 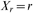 in single populations
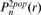	cf. eq. (4)	Probability of 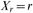 in a 2-population model
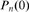	cf. eqs. (5) and (9)	Probability of 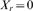 in single populations
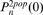	cf. eq. (6)	Probability of 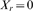 in a 2-population model
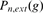	cf. eq. (7)	Extinction probability after 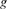 generations of infecteds in single populations
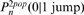	cf. eq. (15)	Probability of 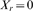 in a 2-population model given that the infectious individual generating secondary cases moves only once between populations
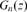	cf. eq. (10)	Probability generating function of 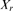
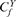	cf. eq. (8)	Sensitivity index equal to the contribution of factor 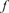 to the variation in 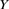

*R.v. = random variable.

All the variables or functions indexed by 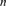 are related to the distribution 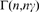 of 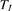.

### Formulation of the Stochastic Model

Analytical calculations and simulations are performed in a continuous-time stochastic framework. Mathematical models used here are classical epidemic models including three distinct disease states and assuming a density-dependent force of infection. The demography is not included, a situation that could fit to rapid infections, spreading and developing on short time scales. The metapopulation context is taken into account by the inclusion of movements between local populations (into and from all compartments) which are considered to have time invariant sizes on average.

Counts of individuals in each patch and for each disease state are represented by random variables defined on a discrete state space. The state of the global system at time 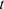 in patch 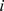 is represented by the vector 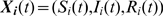, where 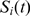, 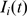 and 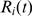 represent the number of susceptible, infectious and recovered individuals respectively.

For the Markovian case, where the probability of future behaviours of the process depends only on the present state, all sojourn times are exponentially distributed. This implies that, for instance, the rate of recovery is constant with respect to time (illustrating the memorylessness of exponential distribution). Transition probabilities for elementary changes in random variables in each patch 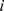 are defined as follows:
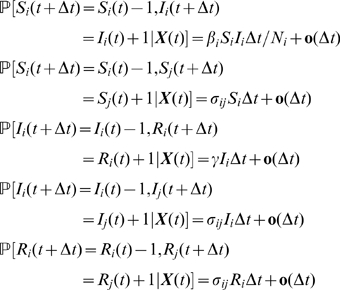
(1)where 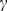 is the recovery rate, 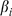 and 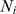 are respectively the transmission rate and the population size of patch 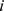 and 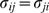 (with 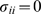) is the per capita rate of migration from patch 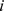 to patch 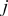.

When the sojourn time in infectious compartment is not exponentially distributed, the associated jump process is no longer Markovian. An appropriate way to deal with this case is to consider the explicit history of each individual rather than a population-based perspective, since in the absence of the lack-of-memory property, removal times of individuals now depend on the time of entering the infective state. In an individual-based formulation, each individual 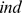 of a population 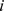 can experience one of the three possible types of transitions during the interval 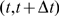:
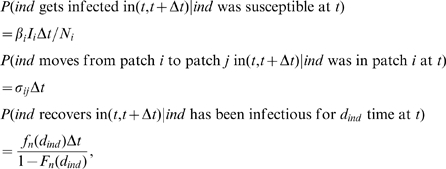
(2)where 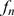 and 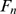 are respectively the probability density and distribution functions of the random variable 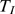 which represents the sojourn time in the infectious state. We take 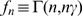 (with integer 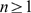) since this formulation embraces the three distributions of 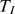 under study: exponential (for 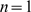), constant (when 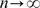) and gamma (for 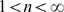). This last case could correspond to a situation where the infectious state of a disease consists in 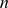 distinct stages, which can be differentiated based on distinct symptoms. Indeed, the duration of a process decomposed in a sequence of 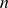 independent stages can be modelled by a 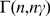 distribution (which can be written as the sum of 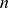 independent exponentially distributed random variables of parameter 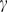). This restriction to integer values for the shape parameter 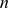 (which corresponds to the Erlang distribution) is very useful in modelling since it captures real scenarios. The mean of the infectious period is the same for all distributions, 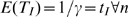.

### Definition of Probabilities of No Secondary Cases in Single and Two Coupled Populations and of Extinction after 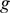 Generations of Infecteds in Single Populations

The probabilities of producing no secondary cases related to early extinction and of extinction after 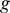 generations summarize the main interesting dynamical behaviours, since they provide information on extinction at different stages in the epidemic evolution. They also allow the comparison between models incorporating gamma distributed infectious sojourn times (with exponential distributed and constant infectious periods are the two extremes) with equal means and with different variances. Prior to performing the comparison of these probabilities provided in the results section, we define them in the context of single and two-coupled populations.

Similarly to Keeling and Grenfell [3], the individual level perspective is considered by introducing the random variable 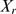 representing the number of secondary cases generated by an infectious individual (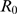 is then the expected value of 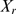). Subscripts indicating the population (as in eq. (1)) are not used in order to avoid overloading notations. According to [3], the probability of generating 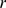 secondary cases by any infectious individual, depending on the shape parameter 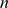 of 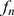, is given by the expression:
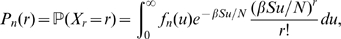
(3)if we assume that new infections are realisations of a homogeneous Poisson process and are produced at a constant rate 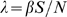. That is, the proportion 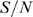 of susceptible individuals remains constant at the beginning or for the duration of an epidemic, which could be generally not so unrealistic for large populations.

In the case of two coupled populations, the production of new cases in each patch is assumed to follow homogeneous Poisson processes of intensities 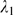 and 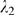 respectively. Let us also define 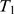, the random variable representing the time spent by an individual in the first patch (assumed to be his origin) during his infectious period 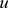, over all successive sojourns. Given 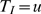, 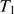 is mixed, since it consists of a mass at 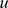 (associated with the Dirac mass equal to 1 in 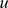 and to 0 elsewhere) and an absolutely continuous component on 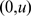. The mass component, noted by 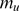, corresponds to the case where the individual does not leave his initial patch. The continuous component, noted by 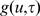, is a continuous function of variables 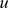 (the infectious duration of the individual) and 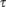 (the part of 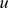 spent in population 1). We also introduce 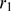, the variable representing the number of secondary cases that this individual generates in the first patch, given that he globally produces 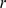 secondary infections. Then, the probability of generating 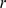 secondary cases in two coupled populations by any infectious individual moving from one population to the other, depending on 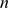 and noted by 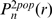 is defined by:
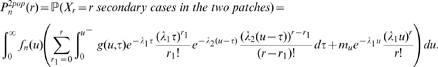
(4)From eqs. (3) and (4), probabilities of generating no secondary cases (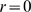), are obtained for single and two coupled populations:
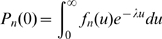
(5)and
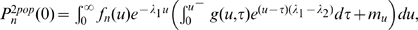
(6)respectively, where 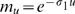 and 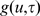 has to be calculated.

Another criterion for comparing the impact of 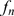 shape on epidemic dynamics is the extinction probability after 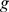 generations, if the production of new cases is interpreted as a branching process. Similarly to [3], this probability can be expressed in a single population under a recursive form:
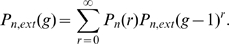
(7)


### Ingredients for a Sensitivity Analysis of the Outputs of Epidemic Dynamics in Metapopulations

Analytical investigations become less tractable for more than two populations, especially for situations where the connections between populations are not homogeneous, i.e. not all populations are connected or migration intensities are different between couples of populations. Moreover, the matter of extinction in metapopulations was explored by simulations elsewhere [1].

In order to explore more broadly the interplay between infectious period shape and other factors (such as 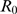 and network connectivity) in terms of impacts on metapopulation dynamics, a sensitivity analysis evaluating how model outputs vary when entries are modified is performed. Special attention is paid to the potential influence of the network (mainly in terms of mean connectivity) through which populations are connected.

The sensitivity analysis is conducted through an analysis of variance (ANOVA). In this kind of analysis, the variance of each *dependent variable* is partitioned into components due to different *factors* in order to see if the variability observed in the dependent variable is due to variations in factors or results from “by-chance” effects. *Factors* are independent variables whose values are controlled and varied by the experimenter. *Dependent variables* represent the response that is observed as a consequence of the independent variables being manipulated.

In the following, details are given on the input *factors*, the *dependent variables* and the formulas used to assess the effect of each factor.

Several input *factors* are tested: (i) the shape of the infectious period distribution, (ii) 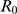, (iii) the transmission rate (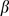), (iv) the mean infection duration (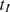), (v) the migration rate (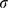) and (vi) the network topology.

Three special shapes for the infectious duration distribution belonging to gamma family are considered: 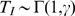 (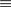
Exp(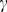); exponential), 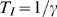 (constant) and 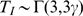 (gamma).

For each of the remaining input factors, several values (reported in Table 2) are chosen to illustrate a large panel of possible realistic situations in terms of capacity of transmission, average duration of infection as well as coupling and connectivity between populations. In these scenarios, 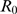 (calculated as 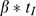) lies on a range from 0.4 to 9.

**Table 2:** Values taken by input factors in ANOVA.

*Parameters*	*Definition*	*Values*
Distribution	Infectious period distribution	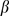 Exp( 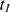 ), 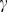 , 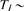
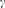 (day 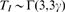 )	Transmission rate	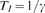
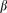 (day)	Mean infection duration	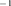
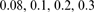 (day 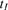 )	Intensity of migration	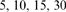 × 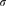 × 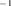
Network	Network topology	Complete, Ring, Star, Homogeneous-random (6, 10, 20, 30, 40, 50)*, Scale-free (6, 10, 20, 30, 40, 50)*

All the values for the 4-uples (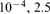, 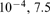, 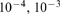 and Network) were considered for each of the three shapes of the infection duration distribution (960 different scenarios). In ANOVA all these factors were treated as ordinal variables by transforming numerical values in ordered categories.

*Numbers in brackets represent mean connectivities of networks that were tested.

Several networks structures are proposed in order to test the influence of the connectivity structure, both in terms of mean connectivity and degree distributions (since not all nodes in a network have the same number of connections). These two characteristics are known to be particularly important for mechanisms by which diseases spread over networks [26]. First, the networks tested here are chosen to represent a large panel of topologies according to the two mentioned features. They are the ring, the star, the completely connected network, the homogeneous-random network and the scale-free network. For our metapopulation comprising 100 population (or nodes) the mean connectivity is equal to 99 for the completely connected network, 2 for the ring network and 1.98 for the star network. For the homogenous-random and scale free networks several average degrees of connectivity were tested: 6, 10, 20, 30, 40 and 50. Second, for a given mean connectivity, the networks tested are also different according to the distribution of their degree of connectivity: e.g. a homogenous-random network is characterized by a Poisson distribution, whereas a scale-free network has a power-law tail and comprises both highly connected hubs and very low connected nodes [26].

All the scenarios obtained by crossing the values given in Table 2 are simulated: 960 scenarios are tested for each of the three distributions of the infectious period. For each scenario, the parameters are taken equal for all populations.

The following *dependent variables* (represented here by *epidemic outputs*) are considered for each simulated scenario corresponding to a fixed set of parameters and many simulation runs: (i) the proportion of minor epidemics, (ii) the epidemic duration, (iii) the date of intra-population epidemic peak averaged over all the populations of the network, (iv) the date and (v) the size of the epidemic peak of the metapopulation (considered as a whole) and (vi) the final epidemic size. All but (i) criterion are calculated for major epidemics only. Minor epidemics (assimilated to the probability of early extinction) and major epidemics (i.e. epidemics that do not undergo early extinction) are distinguished according to the proportion of population that is finally infected. More precisely, for each scenario 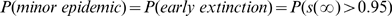 (where 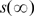 represents the proportion of susceptibles in the total population at the end of the outbreak) and is calculated as 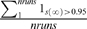 (where 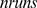 is the number of simulation runs by scenario). Similarly, major epidemics correpond to epidemic trajectories which satisfy the constraint 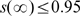.

Criteria (iv), (v) and (vi) are calculated at both individual and population levels. For each scenario and for all but (i) dependent variables, means and variances over major epidemics among simulation runs are calculated.

Since we focus our interest on the shape of the distribution of the infectious period, we separately consider this factor. Therefore, for each scenario, three variants are considered for each dependent variable except for the proportion of minor epidemics: 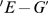 (difference between the value of the output simulated with the exponential distributed infectious period and the value corresponding to the gamma distributed infectious period), 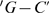 (difference between the value of the output simulated with the gamma distributed infectious period and the value corresponding to a constant infectious period) and 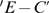 (difference between the value of the output simulated with the exponential distributed infectious period and the value corresponding to a constant infectious period).

The global contribution of *factor* 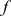 to the variations in the *dependent variable* 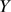 is assessed through a sensitivity coefficient, 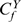, including the principal effect and first-order interactions in which factor 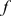 is involved [27]:
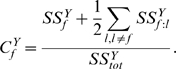
(8)In eq. (8) 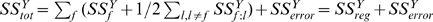 is the total sum of squares (i.e. the sum of squared distances from any point in the dataset to the mean of the data) for the dependent variable 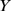, 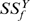 is the sum of squares related to the principal effect of factor 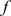 on 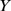 and 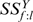 represents the sum of squares related to the effect of interactions between factors 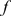 and 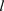 on 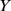. In our analysis, one ANOVA is carried out separately for every dependent variable 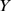 which corresponds to each of the three variants (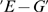, 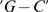 and 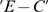) of epidemic outputs previously described. 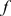 corresponds to each of our input factors. Logarithmic transformation is used for the dependent variables. This or other appropriate transformations of dependent variables are sometimes needed to render linear their relationships with factors, in order to fulfil one of the main assumptions of standard ANOVA (which assumes a linear regression).

In order to more specifically identify factors influencing the proportion of minor epidemics (accounting for early extinction) we also calculate partial rank correlation coefficients (PRCC) for this variable. PRCCs allow the quantification of the non linear association between a given input and the dependent variable when controlling for the other input parameters.

Simulations are performed using a model including 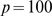 populations of 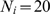 individuals each, coupled by identical migration rates (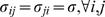) and with equal transmission rates (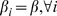). The initial condition is generated by randomly seeding 5 of 100 populations of the network (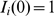 and 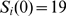 for 5 of 100 populations and 0 and 20 respectively for all remaining ones; 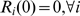). For each scenario, 300 simulation runs are performed using a time continuous event-driven approach based on eqs. (1) and (2) and including three categories of possible events: (i) infection of a susceptible individual (to which corresponds an exponential distributed sojourn time in compartment 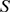 of parameter 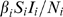), (ii) recovery of an infectious individual (to which corresponds exponential, gamma distributed or constant sojourn times in compartment 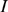 of parameters 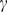, 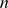 and 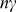 and 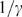 respectively) and (iii) migration of an individual between populations regardless of his disease state (to which corresponds an exponential distributed sojourn time in the population of origin, 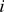, before leaving for population 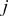, of parameter 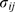). In the case where all transitions are Markovian we use the classical Gillespie's algorithm (details in [1], p201) that simulates the time until the next event and its type. For gamma distributed or constant infectious duration, when the transitions from infectious to recovered states are no longer Markovian, it is necessary to explicitly simulate the history of each individual instead of using the Gillespie's algorithm. In this individual-based approach, the time until the next event considering the metapopulation as a whole will be the minimum over all the individual times. Algorithms are implemented in C language. The various network topologies underlying the metapopulation are generated using *sispread* software [28].

## Results

### Comparison of Probabilities of No Secondary Cases and of Extinction after 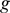 Generations of Infecteds in Single Populations with Respect to the Infectious Period Distribution

First, we expanded the work of Keeling and Grenfell [3] who treated the case of constant distributions for one population, to the gamma distributed sojourn times. We then extended our analysis to the case of two populations.

For a gamma distributed infectious period 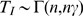 (i.e. 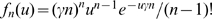, where 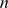 takes positive nonzero integer values), eq. (3) becomes:
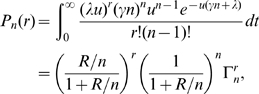
(9)where 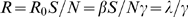 is the effective reproductive number and 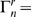 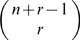 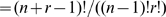. This generalizes the expressions found in [3] for the cases where 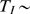 Exp(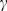) and 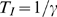 which can be recalculated from eq. (9) by making 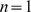 and 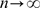, as 
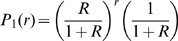
 and 
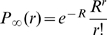
 respectively.

The probability of generating no secondary infections, 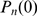, defined in eq. (5), can be viewed as a particular case of the probability generating function (p.g.f.) of 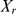. Let 
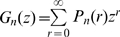
 (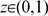) denote the p.g.f. of 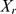 when 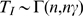. Using eq. (9) 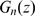 becomes:
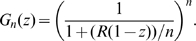
(10)For any 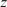 in 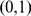, 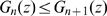 (the inequality is strict unless 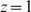). This is easily shown by considering the continuous function 
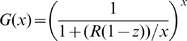
 which has a negative derivative 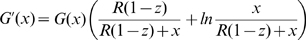 for any 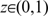. Moreover, 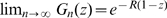 which corresponds to the p.g.f. of 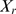 when the infectious sojourn time is constant. As 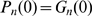, we have, 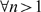:
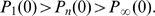
(11)This inequality implies that, for a given individual, the probability of producing no secondary infections is the greatest if the sojourn time in the infectious compartment follows an exponential distribution, which means that in this case the infection is more prone to extinction in early phases.

An order relation can be also established on 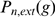 defined in eq. (7). As 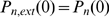, following eq. (11), we have more generally that 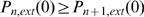, 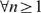. By recurrence, we can show that this inequality holds for all 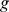. We assume that this statement is true for 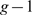 and will prove that it is also true for 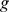. Indeed, by using the fact that, for any 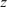 in 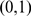, 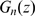 is strictly decreasing in 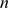 (unless 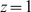) we have that: 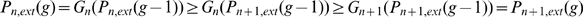. This inequality, suggesting that the exponential model drives more often to extinction even in long term, also holds for the ultimate extinction (whose probability is, for a Galton-Watson process, equal to the smallest positive root of 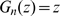). This corroborates the well known result postulating the decrease of the extinction probability as 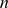 increases.

### Comparison of Probabilities of No Secondary Cases in a System of Two Coupled Populations with Respect to the Infectious Period Distribution

The main scope of this section is to derive a simpler expression for eq. (6), at least for special cases. In a more general context, Takacs [29] provided the distribution of the total time spent in one given state during the time interval 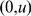 for a process which is assumed alternating between two states. The case where the process is Markovian is treated as a particular case in [29]. Then, 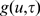 can be calculated as 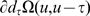, where 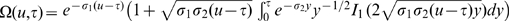 is given in [29], with 
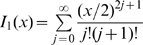
 the modified Bessel function of the first kind of order 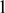.

Instead of differentiating 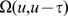 we prefer to provide a more direct and intuitive way to calculate 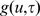. If the number of jumps from one population to the other, which can be odd (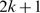) or even (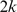), is taken into account, we can write 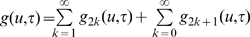. Let us explain the case where the number of switches between populations is odd (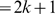), the individual spending 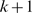 time intervals in each patch. If the respective durations of these time intervals are 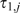 and 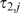 (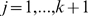), their joint probability density function is:
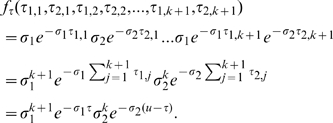


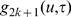
 is obtained by integrating over all possible values of 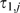 and 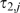:
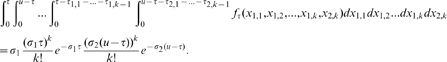
(12)The case where the number of jumps is even is inferred in a similar manner:
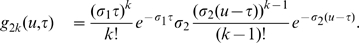
(13)Finally, by summing up eqs. (12) and (13) over all values of 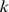 we obtain:
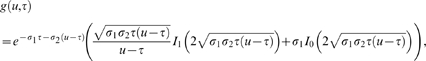
(14)where 
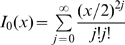
 is the modified Bessel function of the first kind of order 0.

When replacing eq. (14) in eq. (6), the expression of 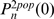 is too cumbersome for allowing the comparison with respect to different shapes of 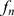. In order to circumvent this problem, the comparison is first explored numerically and then performed analytically on simplified but realistic cases.

**Figure 1:**
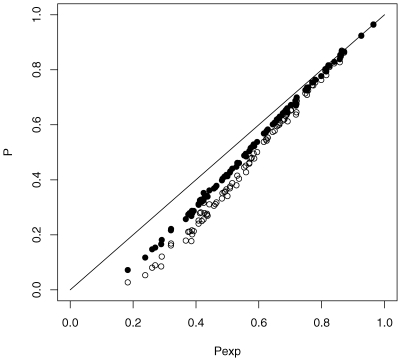
Comparison of probabilities of no secondary cases in a two-population model. On y-axis 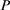 refers to 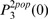 (closed circles) and 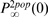 (open circles). On x-axis Pexp refers to 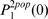. For each of the three probabilities, 100 points with different parameter combinations were generated (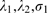 and 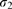 were drawn from exponential distributions and 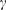 was taken equal to 1). The points represent means over 100000 Monte-Carlo simulations of a time continuous event-driven approach: one infectious individual is introduced in one population and the probability of no secondary cases is calculated based on the time spent in each population. Estimated average standard deviation for computed values was below 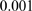. All parameters and variables are explained in Table 1.

We calculate 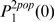 (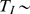
Exp(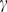)), 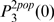 (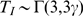) and 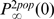 (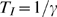) for 100 distinct sets of parameter values (for 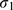, 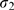, 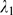 and 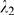). Each value of 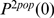 in Figure 1 represents the mean over 100000 Monte-Carlo simulations of a time continuous event-driven approach. According to our simulations, 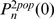 decreases with 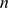 for most of the parameter combinations tested. Nonetheless, the graphic also suggests the existence of regions in the parameter space where 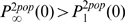 (both probabilities being close to 1). The following two special cases illustrate each of these situations.

Let us consider the case where the individual cannot transmit the disease within his initial population (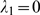) where he cannot return (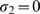) once he left. If we take 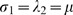, we have 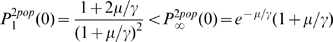 for all 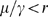, where 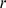 is the root of 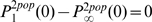.

**Figure 2:**
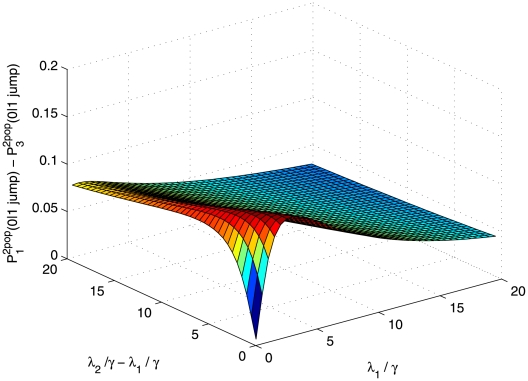
Comparison of probabilities of no secondary cases in a two-population model where individuals move only once. Variation of 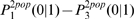 is represented as a function of 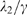 and 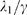 which vary on plausible ranges of values, under the constraint 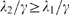. All parameters and variables are explained in Table 1.

Another example practically relevant corresponds to a situation where the number of movements between patches is fixed and equal to one and there is the same probability to switch between patches (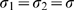). This configuration could correspond to a metapopulation of farms, where animals do not change their original location, unless their are sold (or bought), events that generally occur only once during their lifetime. In this specific case, 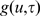 is replaced by 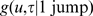 and can be calculated using Bayes' formula as 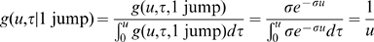 When replacing this expression in eq. (6), where 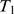 is reduced to its continuous component only, we obtain:
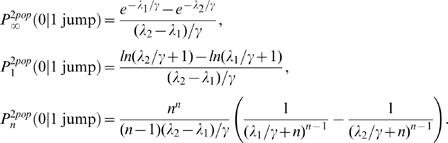
(15)

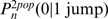
 can also be viewed as the mean of the random variable 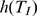, where 
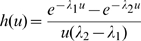
. Indeed, 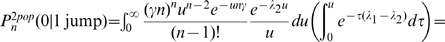 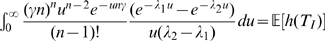. According to Jensen's inequality applied to the convex function 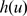, we obtain that 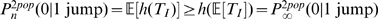. For 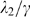 and 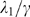 varying on a plausible range of values we have that 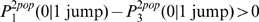, as illustrated in Figure 2. In this case 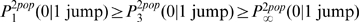.

Contrary to single homogeneously mixing populations, where the probability of generating no secondary cases always decreases as 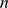 increases, in the case of two coupled populations the monotony of 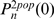 depends on parameter values: it is increasing when 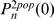 is close to 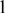 and decreasing elsewhere.

### Examples of Simulated Epidemic Dynamics in a Metapopulation

As an example, here we present for comparison graphical results for a complete graph and a scale-free network with mean degree of connectivity equal to 10. These results illustrate the impact of the mean connectivity and the distribution of the degree of connectivity on the outputs of an epidemic spreading on the network, since the two networks selected are different with respect to these two factors. For each of the three models based on different infectious period distributions, 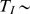
Exp(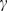) (exponential), 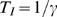 (constant) and 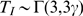 (gamma), with equal mean 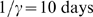 (corresponding to 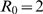), simulations were performed for 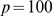 populations of 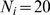 individuals each, coupled by identical migration rates (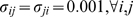) and characterized by equal transmission rates (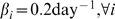). Five of the 100 populations were randomly seeded with 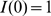.

**Figure 3:**
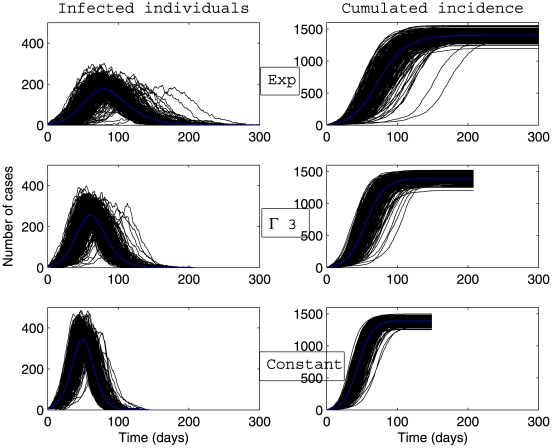
Dynamics of global number of cases and cumulative incidence described by a stochastic metapopulation model based on a completely connected network. Early extinct trajectories were not considered. The mean (blue curve) was calculated over major epidemics only (corresponding to a final attack rate greater than 5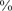). Simulations are performed using a time-continuous event-driven approach with 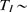
Exp(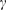) (top panel), 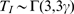 (middle panel) and 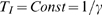 (bottom panel). Parameters values are given in the subsection Examples of Results.

**Figure 4:**
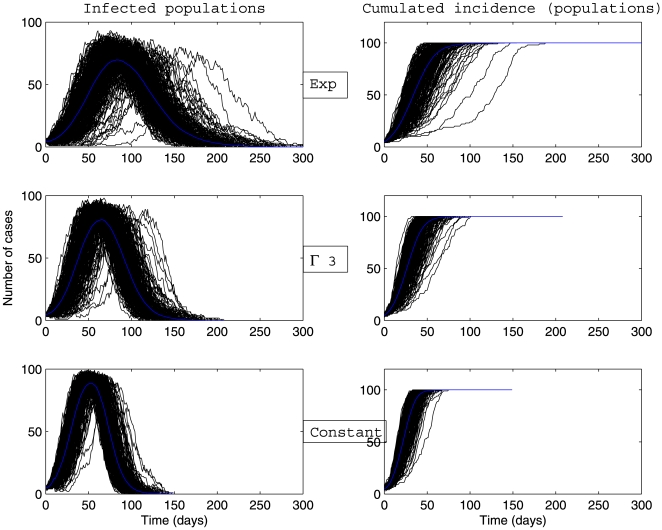
Dynamics of infected populations and cumulative incidence (in number of populations) described by a stochastic metapopulation model based on a completely connected network. Early extinct trajectories were not considered. The mean (blue curve) was calculated over major epidemics only (corresponding to a final attack rate greater than 5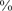). Simulations are performed using a time-continuous event-driven approach with 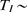
Exp(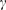) (top panel), 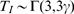 (middle panel) and 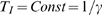 (bottom panel). Parameters values are given in the subsection Examples of Results.

**Figure 5:**
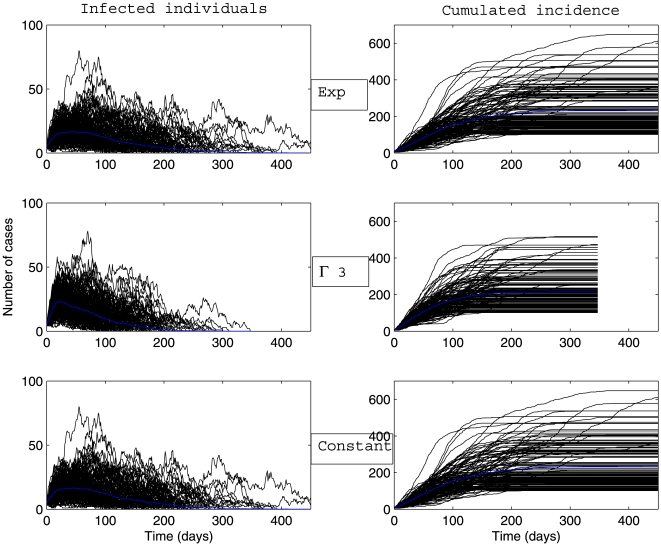
Dynamics of global number of cases and cumulative incidence described by a stochastic metapopulation model based on a scale-free network with mean degree of connectivity equal to 10. Early extinct trajectories were not considered. The mean (blue curve) was calculated over major epidemics only (corresponding to a final attack rate greater than 5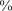). Simulations are performed using a time-continuous event-driven approach with 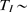
Exp(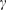) (top panel), 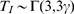 (middle panel) and 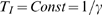 (bottom panel). Parameters values are given in the subsection Examples of Results.

**Figure 6:**
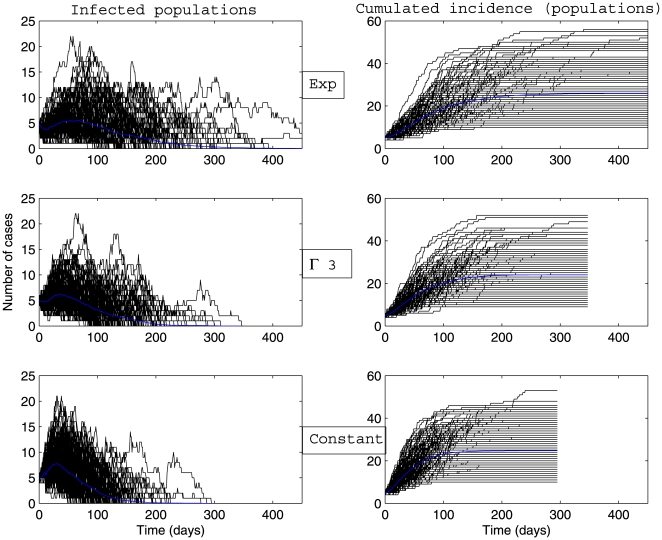
Dynamics of infected populations and cumulative incidence (in number of populations) described by a stochastic metapopulation model based on a scale-free network with mean degree of connectivity equal to 10. Early extinct trajectories were not considered. The mean (blue curve) was calculated over major epidemics only (corresponding to a final attack rate greater than 5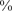). Simulations are performed using a time-continuous event-driven approach with 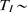
Exp(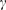) (top panel), 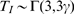 (middle panel) and 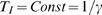 (bottom panel). Parameters values are given in the subsection Examples of Results.

**Figure 7:**
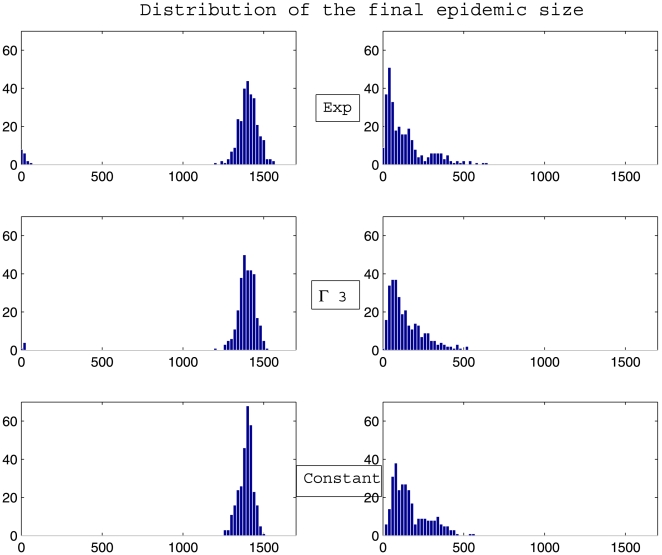
Distribution of the final epidemic size. Calculation was performed on 300 simulations of a stochastic metapopulation model based on a completely connected graph (left panel) and on a scale-free network with mean degree of connectivity equal to 10 (right panel), with 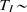
Exp(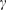) (top graphs), 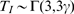 (middle graphs) and 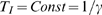 (bottom graphs). Parameters values are given at page 10.

Whatever the scenario considered (Figures 3, 4, 5, 6) and for the parameter values used here, there is a high variability in incidence at the individual and the population levels. Epidemic spread is slowed down by the random nature of the migration between populations. This effect is increased when the mean connectivity decreases (Figures 5, 6). Regardless of the network structure, noteworthy differences between dynamics with respect to the distribution of infectious sojourn time are obtained for the amplitude and the date of the epidemic peak and the epidemic duration. Peaks of the mean incidence at individual and population levels decrease from 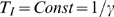 to 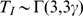 and to 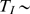
Exp(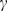). In contrast, the epidemic duration and the variability in incidence increase from the constant to the exponential distributed sojourn times in infectious status (left graphs of Figures 3, 4, 5, 6). For both network topologies in our example, the maximal epidemic duration roughly doubles between the constant and the exponential distributed infectious time based models. The main difference between the networks tested, which could potentially interfere with the influence of the infection time distribution, is illustrated in Figure 7. This figure represents the empirical distribution of the final epidemic size calculated on the 300 stochastic simulations for each scenario. The proportion of minor epidemics is significantly lower in the completely connected graph compared to the low connected scale-free network (right and left panels of Figure 7 respectively), since this latter network facilitates the occurrence of early fade-outs. Moreover, this probability decreases from exponentially distributed to constant infectious durations for both networks. This tendency is more marked for the scale-free network.

### Sensitivity Analysis of the Outputs of Epidemic Dynamics in Metapopulations

**Figure 8:**
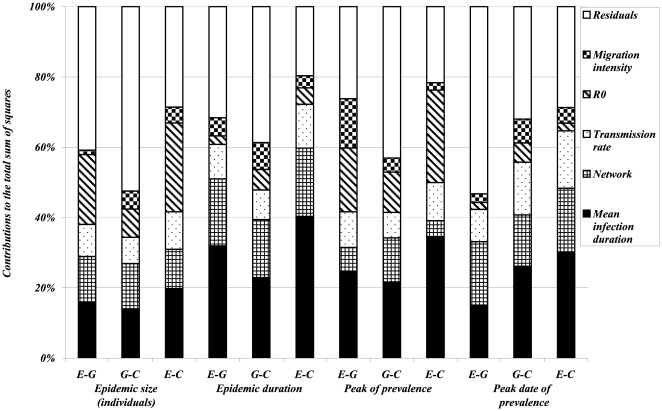
Results of ANOVA on 960 simulated scenarios of epidemic spread with parameter values given in Table 2. *Dependent variables* (on x-axis) are *logarithm of means* (over the non early extinct dynamics) of global variables (directly referring to individuals regardless of their population of origin): *size and duration of the epidemic*, *size and date of the epidemic peak*. For each of these outputs three variants are considered with respect to the distribution of infection duration: 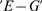 (difference between the value of the output simulated with the exponentially distributed infectious period and the value corresponding to the gamma distributed infectious period), 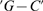 (difference between the value of the output simulated with the gamma distributed infectious period and the value corresponding to a constant infectious period) and 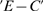 (difference between the value of the output simulated with the exponentially distributed infectious period and the value corresponding to a constant infectious period). Different pattern fills correspond to contributions of five input factors (*mean infection duration*, *network*, *transmission rate*, 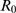 *and migration intensity*) to the variation in outputs amongst scenarios.

**Figure 9:**
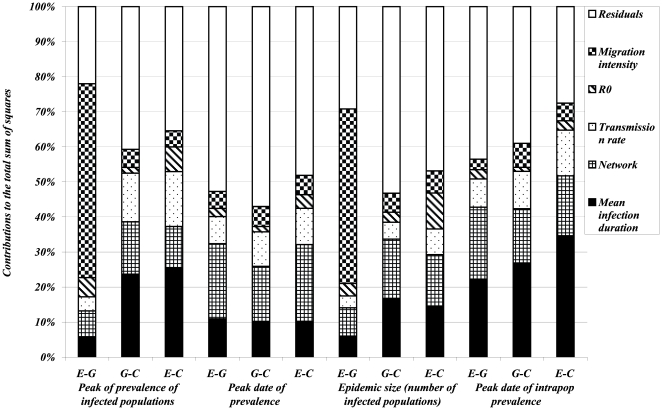
Results of ANOVA on 960 simulated scenarios of epidemic spread with parameter values given in Table 2. *Dependent variables* (on x-axis) are *logarithm of means* (over the non early extinct dynamics) of global variables (referring to populations): *size and duration of the epidemic*, *size and date of the epidemic peak*. For each of these outputs three variants are considered with respect to the distribution of infection duration: 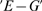 (difference between the value of the output simulated with the exponentially distributed infectious period and the value corresponding to the gamma distributed infectious period), 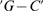 (difference between the value of the output simulated with the gamma distributed infectious period and the value corresponding to a constant infectious period) and 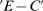 (difference between the value of the output simulated with the exponentially distributed infectious period and the value corresponding to a constant infectious period). Different pattern fills correspond to contributions of five input factors (*mean infection duration*, *network*, *transmission rate*, 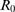 *and migration intensity*) to the variation in outputs amongst scenarios.

**Figure 10:**
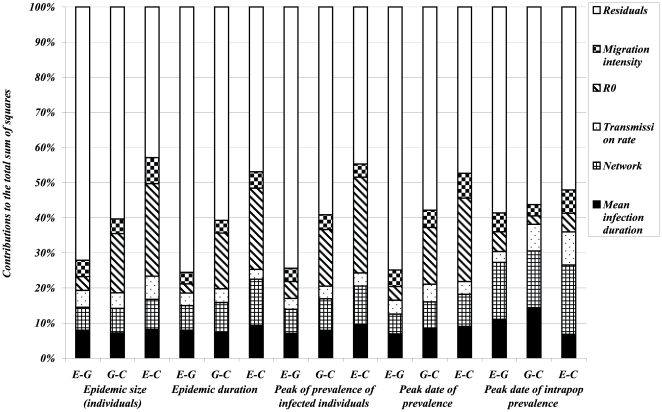
Results of ANOVA on 960 simulated scenarios of epidemic spread with parameter values given in Table 2. *Dependent variables* (on x-axis) are *logarithm of variances* (over the non early extinct dynamics) of global variables (directly referring to individuals regardless of their population of origin): *size and duration of the epidemic*, *size and date of the epidemic peak and date of intra-population epidemic peak*. For each of these outputs three variants are considered with respect to the distribution of infection duration: 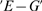 (difference between the value of the output simulated with the exponentially distributed infectious period and the value corresponding to the gamma distributed infectious period), 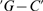 (difference between the value of the output simulated with the gamma distributed infectious period and the value corresponding to a constant infectious period) and 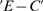 (difference between the value of the output simulated with the exponentially distributed infectious period and the value corresponding to a constant infectious period). Different pattern fills correspond to contributions of five input factors (*mean infection duration*, *network*, *transmission rate*, 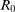 *and migration intensity*) to the variation in outputs amongst scenarios.

As illustrated by simulations described in the previous section, the choice of the distribution of the infection duration has various impacts on global epidemic outputs such as epidemic duration, epidemic size or epidemic peak in a metapopulation. In addition, these impacts could vary with respect to other parameters. The sensitivity analysis provides a quantification of these potential interactions. Namely, we explore whether predominant contributing factors are the same for every output of the epidemic and also if they are identical for the three variants 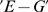, 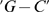 and 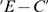 of a given output. Results are summarized in Figure 8, 9 and 10: each bar is decomposed in segments with heights equal to the sensitivity coefficients, which evaluate the percentage of the global variability of the dependent variable explained by each factor.

In order to explicitly illustrate our sensitivity analysis approach, let us describe, as an example, the statistical analysis performed on the epidemic size. For a given set of parameters we simulate epidemics with exponentially, gamma distributed and constant infectious periods and calculate means and variances of their epidemic sizes, noted by *MESexp*, *MESgamma*, *MESconst*, *VESexp*, *VESgamma*, *VESconst* respectively. To account for nonlinear relationships between outputs and factors we apply the log transformation to outputs. Then, a classical ANOVA is performed on each couple of differences, as dependent variables, *(log(MESexp)-log(MESgamma))*, *(log(MESgamma)-log(MESconst))*, *(log(MESexp)-log(MESconst))*, *(log(VESexp)-log(VESgamma))*, *(log(VESgamma)-log(VESconst))* and *(log(VESexp)-log(VESconst))*. For each of these dependent variables, sensitivity coefficients are then calculated using the equation (8) for each of the factors of interest (Figures 8, 9, 10). Let us focus on differences between exponential and gamma based models. For instance, according to Figure 8, we can say that 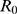 is the most influential factor impacting on the difference in the mean epidemic expressed in terms of infected individuals. In other words, the error in the prediction of the mean epidemic size committed if an exponential distribution was used instead of more realistic gamma distribution for the infectious duration heavily depends on 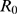. At a population scale, the most important factor for the epidemic size is the migration intensity, whereas differences in variances are almost equally and weakly impacted by all factors.

More generally, when analyzing means over major epidemics of outputs directly referring to individuals, the main factors explaining their variations are first the mean infection duration and second the network structure or 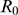, depending on the output of interest (Figure 8). The migration intensity contributes the least to the majority of outputs. This order of importance is preserved for most outputs whatever the distributions compared (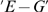, 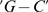 and 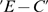). For outputs directly referring to the population level (Figure 9), the mean infection duration and the network structure are the most important factors for the majority of outputs. As an exception, the migration intensity plays the major role for the 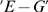 variant of the epidemic size and of the peak of prevalence. The outputs expressed as variances over all simulated major epidemics (Figure 10) are less influenced by input factors. For the majority of outputs the most contributing factors are 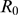, the network structure, and the mean infection duration. It is noticeable that the variance of 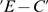 variant of nearly all outputs is better explained (higher coefficients of determination 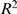) by input factors than 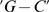 and 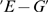 variants.

Regarding the early extinction, the most important correlations of the proportion of minor epidemics are with 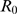 (PRCC = −0.57), the migration intensity (PRCC = −0.48) and the network (PRCC = −0.36). The metapopulation epidemic has more chances to go extinct as 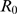 decreases and as populations are less connected. The distribution of the infectious period is more weakly correlated with the proportion of early extinct dynamics (PRCC = 0.13).

## Discussion

In this paper, we studied the impact of the infectious period distribution on the global dynamics described by a metapopulation model comprising many patches. Since assuming exponential sojourn times in infected states is a common approximation used in most of mathematical models, we were interested in evaluating the potential bias that such an assumption would introduce at a global scale of an epidemic. Besides some analytical developments extending the work of Keeling and Grenfell [3] in single populations, we deliberately focused our attention on metapopulations. There are two main reasons for this choice. First, the impact of the infection period distribution in single populations was extensively explored in the literature ([2], [3] and many other studies, especially in the context of HIV epidemic). Second, we were interested in exploring the potential interactions between effects of the infection duration distribution and of exogenous factors (such as the topology of the network underlying the metapopulation, or the migration intensity), elements which are not present in single populations.

First, we performed analytical investigations. As mentioned above, we extended developments of [3] for single populations to the case of gamma distributed infectious durations, and showed that the probability of extinction in early phases increases with the variance of the infectious duration (which is the largest for exponential distribution, given the same mean for all distributions and 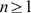 for 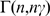). This result corroborates what it was shown for branching-type epidemic processes with an 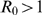 [2]. The same order in the extinction probabilities holds after 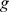 generations of infecteds.

The case of two coupled populations was also explored through a similar approach invoking Poisson processes for the generation of secondary infection and Markovian transitions for the migration between patches. Under the constraint that an individual changes only one time his patch of residence during the duration of his infectious period, a plausible assumption for real animal populations, we showed that here again the probability of producing no secondary cases is the greatest when 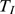 is exponentially distributed. Inversely, we identified special cases where the probability of early extinction in a two-population system increases as 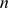 increases. This occurs, for instance, under a specific constraint on parameters, in a situation where the individual cannot transmit the disease within his initial population where he cannot return once he left.

Therefore, caution has to be paid when interpreting extinction results provided by mathematical models: the extinction probability could be over or underestimated in situations where the exponential distribution is particularly not appropriate for modelling the infection duration. This could be the case for long lasting diseases where the probability of recovering strongly depends on the disease stage.

Although analytical explorations were performed on 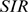 models, they would be completely transposable to the 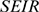 case, as the introduction of a latent period would not affect the quantities analyzed in this study. Besides, some of our results, as those in [3], were rigorously proved based on the assumption of no depletion of susceptibles during the epidemic process. Since this is an acceptable approximation when sizes of target populations are large, it could become awkward to defend it when working with small populations.

In a second part, we focused on a metapopulation context comprising more than two patches and relatively small population sizes, without making the assumption of constant proportion of susceptibles over time. Since analytical explorations were too complex for this case, it was studied by simulations and statistical analysis of simulated data. As already mentioned above, in the metapopulation framework, we were not exclusively interested in the potential effect of the shape of the infection duration distribution on epidemic dynamics. We also explored in which extent this effect could be influenced by other factors, especially those directly related to the metapopulation structure, such as the mean degree of connectivity of the network or the migration intensity between patches.

The impact of network topology on the spread of epidemics was previously explored (see [30] for a review). However, the assumption for the distribution of infection period was neither discussed nor taken into account in those studies. In addition, most often, the nodes of such networks are individuals and not populations and infection spread between patches is indirectly considered through distance-based transmission rates. In our study, both intra-population epidemic dynamics (described by a specific 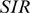 model for each patch) and inter-population spread of infection (taken into account through migration of individuals) were considered.

The sensitivity analysis performed indicated that the most important factors which influence the impact of infection duration distribution on epidemic outputs in metapopulations are 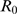, the network structure and the mean infection period. This means that under or overestimation of epidemic outputs such as epidemic duration and prevalence peak size, due to specific modelling choices concerning the distribution of infection duration, depends on other factors related to the infectious potential of the pathogen and to the way the populations are connected. Nevertheless, these effects are not identical for all criteria: for instance, the peak of population prevalence is strongly influenced by the migration intensity (Figure 9), whereas variation in its date is mostly explained by the network topology (Figure 10). A similar statistical analysis conducted within a single population and including only the transmission rate and the mean infections duration as factors (results not shown) reveals that 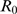 is the most influential factor on size-related outputs (such as epidemic burden or epidemic peak), whereas the mean infection duration preferentially impacts time-related outputs (such as epidemic duration or peak date).

The sensitivity indices and hence the relative importance of each factor in explaining the variability of a given criterion could depend on the number of factors included in the analysis, but their relative ordering should not change.

As a first analysis of the potential effect of the shape of infection duration distribution on global dynamics, all intra-population parameters have been considered consistent among populations of the network. We also considered small intra-patch population sizes (equal to 20). This could correspond to a group of small farms but also to the classes of a school and hence refers to human populations. Further research is needed to investigate heterogeneous metapopulations characterized by unequal contributions of patches to the global spread of a disease and also to rigorously assess the robustness of results to changes in the population size.

We have shown that the effect of assuming exponential distribution for infection periods instead of more realistic distributions varies with respect to the output of interest and to other exogenous factors. Attention has to be paid to all these elements in practice. For example, when evaluating control strategies at a global level by modelling approach in order to optimize their use, expected losses due to the disease spread as well as expected gains due to interventions may be misestimated. Future research should more closely explore under what circumstances the exact distributions are relevant in order to assess in which situation effort should be put into obtaining them.
